# Bortezomib Inhibits Hypoxia-Induced Proliferation by Suppressing Caveolin-1/SOCE/[Ca^2+^]_i_ Signaling Axis in Human PASMCs

**DOI:** 10.1155/2021/5551504

**Published:** 2021-04-08

**Authors:** Chao Wang, Yuanqi Li, Lei Xu, Qiang Zhang, Gegentuya, Guoying Tian

**Affiliations:** ^1^Department of Pulmonary and Critical Care Medicine, The Affiliated Hospital of Inner Mongolia Medical University, Hohhot, Inner Mongolia Autonomous Region, China 010059; ^2^Health Management Center, The Fifth Affiliated Hospital of Guangzhou Medical University, Guangzhou, Guangdong, China 510700; ^3^Department of Dermatology, The Affiliated People's Hospital of Inner Mongolia Medical University, Hohhot, Inner Mongolia Autonomous Region, China 010059; ^4^Department of Image Diagnoses, The Affiliated Hospital of Inner Mongolia Medical University, Hohhot, Inner Mongolia Autonomous Region, China 010059

## Abstract

**Background:**

Previous studies have demonstrated the ubiquitin-proteasome inhibitor bortezomib (BTZ) can effectively alleviate hypoxia-induced pulmonary hypertension (HPH) by suppressing the intracellular calcium homeostasis in pulmonary arterial smooth muscle cells (PASMCs). Further evaluation showed that the antiproliferation roles of BTZ are mainly mediated by inhibition of the intracellular calcium homeostasis. Caveolin-1 belongs to one of the key regulators of the intracellular calcium homeostasis in PASMCs, which can regulate the store-operated calcium entry (SOCE). However, the effects of BTZ on Caveolin-1 remain unclear.

**Methods:**

Primarily cultured human PASMCs were used as the cell model. CCK-8 assay was performed to assess the PASMCs proliferation. Western blotting and real-time qPCR were used to detect the mRNA and protein expressions. Fura-2-based fluorescence imaging experiments were used to determine the intracellular calcium concentration ([Ca^2+^]_i_). The protein synthesis inhibitor cycloheximide (CHX) was utilized to determine the protein degradation process.

**Results:**

Firstly, in cultured human PASMCs, treatment of BTZ for 24 or 60 hours significantly downregulates Caveolin-1 at both mRNA and protein levels. Secondly, in the presence CHX, BTZ treatment also leads to downregulated protein expression and fastened protein degradation of Caveolin-1, indicating that BTZ can promote the Caveolin-1 protein degradation, other than the BTZ on Caveolin-1 mRNA transcription. Then, BTZ significantly attenuates the hypoxia-elevated baseline [Ca^2+^]_i_, SOCE, and cell proliferation.

**Conclusion:**

We firstly observed that the ubiquitin-proteasome inhibitor BTZ can inhibit the Caveolin-1 expression at both mRNA transcription and protein degradation processes, providing new mechanistic basis of BTZ on PASMC proliferation.

## 1. Introduction

Pulmonary hypertension (PH) is a serious cardiopulmonary disease syndrome that can be defined by many pathophysiological features, including the progressive increase of the pulmonary arterial pressure and significant thickening and remodeling of the small pulmonary arteries (PAs). These changes will eventually cause right ventricle hypertrophy and heart failure [1]. In the past decades, therapeutic drugs for the treatment of PH that can induce vasodilation and exert antiproliferation and antimigration effects have been studied, which can relieve the PA vascular remodeling [2]. However, these drugs can only partially improve the disease symptom, but cannot fundamentally cure this deadly disease. So, to search for new class of treatments with better efficacy is important.

It has been well accepted that the substantial vascular remodeling of the small PAs is induced by the hyperproliferation and migration of the pulmonary arterial smooth muscle cells (PASMCs). Previous studies have shown the abnormal elevation of the intracellular free calcium concentration ([Ca^2+^]_i_) plays as a key factor to induce the proliferation and migration of PASMCs. In mechanism, the elevated [Ca^2+^]_i_ is mainly caused by two major pathways: (a) calcium release from the intracellular calcium store that localizes on the membrane of endoplasmic/sarcoplasmic reticulum; (b) extracellular calcium entry through the three types of major calcium channels that localize on the plasma membrane including the voltage-operated calcium channels (VOCCs), receptor-operated calcium channels (ROCCs), and store-operated calcium channels (SOCCs) [3]. Previous studies have well demonstrated that the hypoxia-induced elevation of calcium influx through the SOCCs, termed as the store-operated calcium entry (SOCE), contributes most to hypoxia-elevated [Ca^2+^]_i_ and plays as the master regulator to induce the proliferation and migration subsequences [4, 5]. Moreover, the upregulation of the core SOCC components, transient receptor potential cation channels (TRPCs), is the molecular basis of the hypoxia-elevated SOCE and basal [Ca^2+^]_i_ [4]. Other than TRPCs, the upregulation of the plasma membrane scaffolding protein Caveolin-1 is also proved to play critical role in regulating SOCE, proliferation, and migration of PASMCs [6, 7]. Previous studies have shown that Caveolin-1 also participates in the regulation of SOCCs by direct interaction with TRPC1 and the other SOCC core proteins, including STIM1 and Orai1 [8–11].

Recently, the ubiquitin-proteasome inhibitors, including MG-132 [12], bortezomib (BTZ) [13], and carfilzomib [14], have been reported by several studies to successfully attenuate the disease development of different types of experimental PH animal models, through inhibition of the TRPC-SOCE-[Ca^2+^]_i_ signaling axis in PASMCs [13]. Based on these backgrounds, in this study, we further determined whether and how does BTZ affect the expression of Caveolin-1 in both normoxic and hypoxic PASMCs.

## 2. Materials and Methods

### 2.1. Culture and Validation of Primary Human Pulmonary Arterial Smooth Muscle Cells (PASMCs)

All the experimental procedures were approved by the Animal Care and Use Committee of The Affiliated Hospital of Inner Mongolia Medical University. We followed the experimental methods according to protocols described in previously publication [6, 7]. In details, human primary cultured PASMCs were purchased from ScienCell Research Laboratories, Inc. The cells were cultured in smooth muscle growth medium (Lonza) to 60-80% confluence and serum-starved in smooth muscle basal medium (Lonza) containing 0.3% FBS for 24 hours, then treated with the following reagents: proteasome inhibitor BTZ (10 nM), lysosome inhibitor chloroquine (CHQ, 10 *μ*M), or protein synthesis inhibitor CHX (20 *μ*g/ml), under either normoxic or hypoxic (4% O_2_-5% CO_2_) conditions. Prior to each experiment, the cell purity was ensured by over 90% positive staining of the specific smooth muscle cell marker *α*-actin (Sigma-Aldrich) by immunofluorescence staining.

### 2.2. Real-Time Quantitative Polymerase Chain Reaction (RT-qPCR)

After each specific treatment, the total RNA was isolated by using RNeasy column and RNase-free DNase (Qiagen), according to previous publication [6, 7]. Then, the RNA was reverse transcribed by iScript cDNA synthesis kit (Bio-Rad) with reaction mixture containing 500-1000 ng of total RNA. The RT-qPCR was performed by using QuantiTect SYBR Green PCR Master Mix (Qiagen) in an iCyclerIQ detection system (Bio-Rad) and followed by protocols: 95°C~3 min, 40 cycles at 95°C~5 sec and 60°C~15 sec. The primer sequences for each gene were designed using NCBI primer designing tool. 18S was used as internal house-keeping gene. The specific primer pairs for qPCR were Caveolin-1: sense, 5′-CTACAAGCCCAACAACAAGG-3′; antisense, 5′-CATCGTTGAGGTGTTTAGGGT-3′; 18S: sense, 5′-GCAATTATTCCCCATGAACG-3′; antisense, 5′-GGCCTCACTAAACCATCCAA-3′.

### 2.3. Western Blot

After treatment, the PASMCs were lysed using RIPA buffer (Pierce) supplemented by 5% protease inhibitor cocktail (Sigma-Aldrich). The total protein concentration was determined by BCA kit (Pierce). Homogenates were denatured by adding 150 mM dithiothreitol (DTT) and heating at 95°C for 3 min. Whole cell lysates were separated on SDS-PAGE calibrated with Precision Plus protein dual color molecular weight markers (Bio-Rad). The proteins were then transferred onto 0.45 *μ*m polyvinylidene difluoride membranes (Bio-Rad), blocked with 5% nonfat dry milk (Bio-Rad) in Tris-buffered saline containing 0.2% Tween 20 (TBST), and blotted with primary antibodies against Caveolin-1 (BD Biosciences), p21 (BD Biosciences), LC3B (Cell Signaling Technology), or beta-tubulin (Sigma-Aldrich). The membranes were then washed by TBST×3 and incubated with horseradish peroxidase-conjugated goat anti-rabbit or anti-mouse IgG (Kirkegaard and Perry Laboratories). The bands were detected using an enhanced chemiluminescence system (GE healthcare). Gray density of each image was analyzed by Image J; data were calculated as a ratio of the protein of interest to house-keeping protein beta-tubulin, serving as internal control.

### 2.4. Cell Proliferation Assay

The cell proliferation rate was reflected by CCK-8 assay. Specifically, human PASMCs were seeded in 96-well plates (1 × 10^6^ cells/ml) and cultured for 24 hours (37°C, 5% CO_2_). The cells were then serum-starved and treated with BTZ and/or CHQ under either normoxic or hypoxic conditions. After a 60 h treatment, the medium was replaced by 90 *μ*l of medium plus 10 *μ*l of CCK-8, and the cells were incubated for another 4 hours. Then, the viable cell number was determined by a plate reader (Thermo Scientific™ Multiskan™ FC) at 450 nm wavelength. The cell viability was reflected as a percentage to that of the normoxic control group.

### 2.5. Intracellular Calcium Measurement

The baseline intracellular calcium concentration ([Ca^2+^]_i_) and SOCE were measured by previously reported fura-2 fluorescence-based experiments [15].

### 2.6. Statistical Analysis

All the data were shown as mean ± SEM. The “*n*” means the number of individual experiment. The normal distribution of the data was tested by the Kolmgorov-Smirnov test, and the homogeneity of variance was tested by Bartlett's test. Statistical analyses for pairwise comparison were analyzed by Student's *t*-test. One-way ANOVA was used to calculate the statistical significance of multiple groups. Differences were considered significant when *p* < 0.05. The statistical analysis was performed by using SPSS and GraphPad Prism 8.

## 3. Results

### 3.1. Short-Term Treatment of BTZ Did Not Significantly Affect Caveolin-1 Protein Expression in Human PASMCs

**Figure 1:**
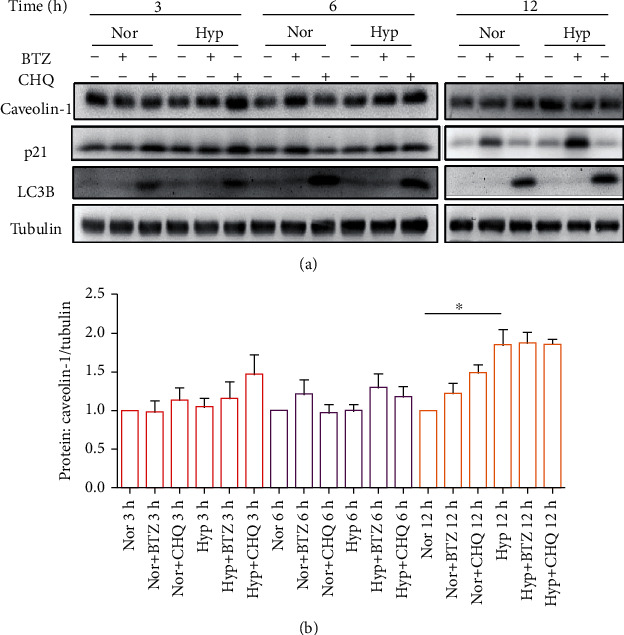
Effects of short-term treatments of bortezomib (BTZ) or chloroquine (CHQ) on Caveolin-1 protein expression in normoxic and hypoxic (4% O_2_) human pulmonary arterial smooth muscle cells (PASMCs). Western blots (a) and bar graph (b) representing the protein expression level of Caveolin-1, p21, and LC3B upon treatment with BTZ (10 nM) or CHQ (*μ*M) for 3, 6, and 12 hours in human PASMCs under normoxic and hypoxic conditions. Beta tubulin was used as house-keeping protein. The bar graph represents the mean ± SEM, *n* = 5 in each group; ^∗^*p* < 0.05, ^&^*p* < 0.05 as indicated.

**Figure 2:**
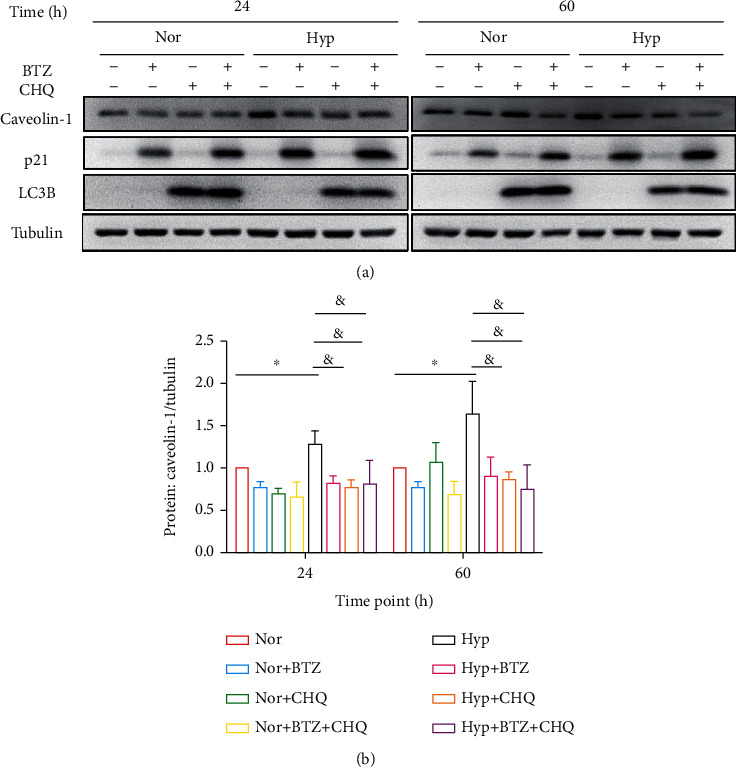
Effects of long-term treatments of BTZ and/or CHQ on Caveolin-1 protein expression in normoxic and hypoxic human PASMCs. Western blots (a) and bar graph (b) representing the protein expression level of Caveolin-1, p21, and LC3B upon treatment with BTZ (10 nM) or CHQ (*μ*M) for 24 and 60 hours in human PASMCs under normoxic and hypoxic conditions. Beta tubulin was used as house-keeping protein. The bar graph represents the mean ± SEM, *n* = 5 in each group; ^∗^*p* < 0.05, ^&^*p* < 0.05 as indicated.

**Figure 3:**
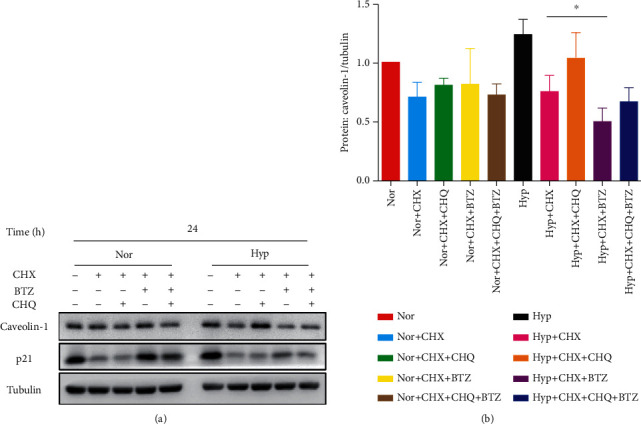
Effects of BTZ and CHX on the Caveolin-1 protein expression in the presence of cycloheximide (CHX) in human PASMCs under normoxia and hypoxia. Western blot (a) and bar graph (b) representing the protein expression level of Caveolin-1 and p21 upon treatment with BTZ and/or CHQ in the presence of CHX (20 *μ*g/ml) for 24 hours in human PASMCs under normoxic and hypoxic conditions. The bar graph represents the mean ± SEM, *n* = 4 in each group; ^∗^*p* < 0.05 vs. Hyp+CHX.

**Figure 4:**
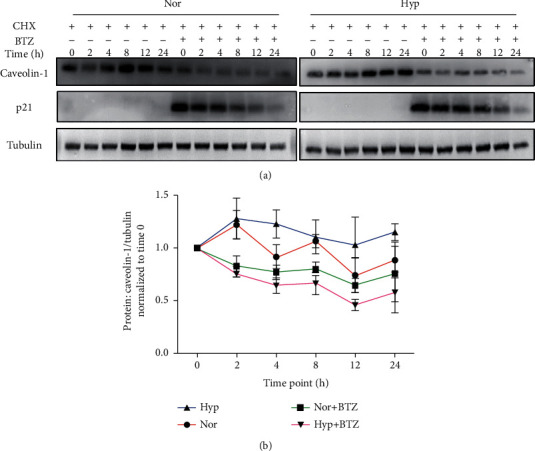
Effects of BTZ on the protein degradation of Caveolin-1 protein in human PASMCs under normoxia and hypoxia. Western blot (a) and line chart (b) representing the protein expression level of Caveolin-1 and p21 upon treatment with/without BTZ in the presence of CHX within a 24-hour time window in human PASMCs under normoxic and hypoxic conditions. The bar graph represents the mean ± SEM, *n* = 5 in each group.

Previous studies have well demonstrated the roles of BTZ in suppressing the intracellular calcium and SOCE by inhibiting the expression of major SOCC components TRPC1 and TRPC6; therefore, we further determined the role of BTZ on another key regulator of SOCC, Caveolin-1. As seen in Figure 1, by performing time-course (3-, 6-, and 12-hour) treatment experiments, our data indicated that a short-term treatment of BTZ (10 nM) did not significantly affect Caveolin-1 protein expression in both normoxic- and hypoxic (4% O_2_)-treated PASMCs. Knowing the fact that Caveolin-1 protein degrades through the lysosome system [16], specific lysosome inhibitor CHQ was used as a positive control, which represents more or less effects to induce increased Caveolin-1 protein expression. To confirm the effective blockage of BTZ on the ubiquitin-proteasome system, we included a positive control protein, p21, which has been widely proved to be degraded through the ubiquitin-proteasome system. As is seen in Figures 1, 2, 3, and 4, a dramatic time-dependent induction of p21 was obtained after BTZ treatment from short- to long-term time points. In parallel, to confirm the effective blockage of CHQ on the lysosome-autophagy system, we also included a positive control protein, LC3B, which has been well shown to be degraded through the lysosome-autophagy system. As is represented in Figures 1 and 2, dramatic time-dependent induction of LC3B was also obtained following the short to long term of CHQ treatment.

### 3.2. Long-Term Treatments of Both BTZ and CHQ Inhibited Caveolin-1 Expression in Hypoxic PASMCs

**Figure 5:**
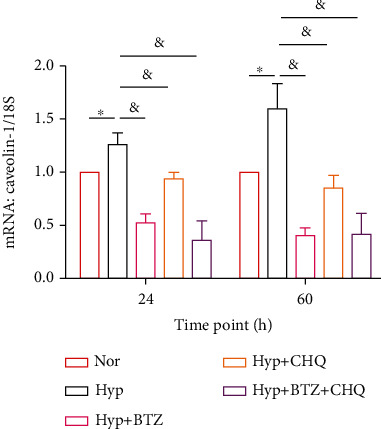
Effects of long-term treatments of BTZ and/or CHQ on Caveolin-1 mRNA expression in human PASMCs under normoxia and hypoxia. Bar graph representing the mRNA expression level of Caveolin-1 upon treatment with BTZ and/or CHQ for 24 and 60 hours in human PASMCs under normoxic and hypoxic conditions. 18S was used as house-keeping gene. The bar graph represents the mean ± SEM, *n* = 4 in each group; ^∗^*p* < 0.05, ^&^*p* < 0.05 as indicated.

To further dissect the mechanisms under the suppression of BTZ on Caveolin-1 expression, we found that a long-term treatment (24- and 60-hour) of BTZ significantly inhibited the hypoxia-induced Caveolin-1 expression at both protein (Figure 2) and mRNA (Figure 5) levels. However, we surprisingly found that a long-term treatment (24- and 60-hour) of the lysosomal inhibitor CHQ (10 *μ*M) also significantly inhibited the protein expression of caveolin-1 in hypoxic PASMCs, which are against the fact of CHQ as a lysosome inhibitor, supposing to induce Caveolin-1 protein accumulation by inhibiting its protein degradation. Nevertheless, these data indeed correlated with previous data showing that treatment of CHQ can effectively attenuate the CPA-induced SOCE in PASMCs [17]. We then checked the effects of CHQ on Caveolin-1 transcription, which showed that a 24- and 60-hour treatment of CHQ induced markedly downregulation of Caveolin-1 mRNA (Figure 5), suggesting the long-term downregulation of CHQ on Caveolin-1 might be due to the inhibition on Caveolin-1 transcription. Moreover, compared to monotreatment with either BTZ or CHQ, a combined treatment of BTZ+CHQ brings no further decrease in Caveolin-1 expression at either protein (Figure 2) or mRNA (Figure 5) levels.

**Figure 6:**
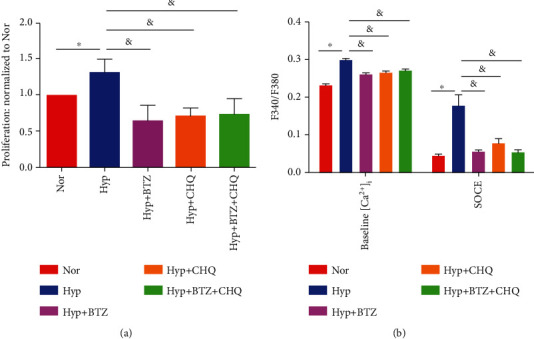
Effects of BTZ and/or CHQ treatment on the proliferation and intracellular calcium homeostasis of human PASMCs under normoxia and hypoxia. Bar graphs representing the normalized proliferation (a), baseline [Ca^2+^]_i_, and SOCE (b) of PASMCs upon treatment with BTZ and/or CHQ for 60 hours under normoxic and hypoxic conditions. The bar graph represents the mean ± SEM, *n* = 6 in (a) and *n* = 3 in (b); ^∗^*p* < 0.05, ^&^*p* < 0.05 as indicated.

In parallel, our data also showed that the monotreatment with either BTZ or CHQ significantly attenuated the hypoxia-induced proliferation in PASMCs, but a combined treatment of BTZ+CHQ brings no add-on inhibition on PASMCs proliferation (Figure 6(a)), baseline [Ca^2+^]_i_, and SOCE (Figure 6(b)), suggesting Caveolin-1/SOCE/[Ca^2+^]_i_ signaling axis may exert as a common pathway for the two classes of reagents BTZ and CHQ.

### 3.3. BTZ Inhibited Caveolin-1 Protein Expression by Promoting Its Protein Degradation through Lysosome System in Both Normoxic and Hypoxic PASMCs

Besides the suppressive roles of BTZ on the transcription of Caveolin-1, we also assessed whether BTZ can affect the Caveolin-1 protein degradation process by introducing the protein synthesis inhibitor CHX (20 *μ*g/ml). As seen in Figure 3, our data showed that in the presence of protein synthesis inhibitor CHX, treatment with the CHQ can partially rescue the CHX-induced decline in Caveolin-1 protein level due to the blockage of lysosomal degradation; while BTZ treatment further accelerated the decline in Caveolin-1 protein in hypoxic PASMCs, suggesting the role of BTZ in promoting Caveolin-1 protein degradation through lysosome system.

To confirm our hypothesis, we then performed a time-point protein degradation experiment, captured the Caveolin-1 protein degradation dynamics by analyzing the percentage of remaining Caveolin-1 protein at 0, 2, 4, 8, 12, and 24 hours after CHX treatment in both control and BTZ (48-hour) treatment cells. As seen in Figure 4, our data represented accelerated protein degradation curve of Caveolin-1 in hypoxia+BTZ treatment group, compared to the hypoxic control group. These data collectively suggest that other than the suppression of BTZ on Caveolin-1 transcription, BTZ can also promote the protein degradation of Caveolin-1 through the classic lysosome degradation pathway.

## 4. Discussion

The caveolae regions are a domain of invaginations that locate at the plasma membrane and function in a list of intra- and extracellular communication events, such as the extracellular calcium influx, endocytosis, and extracellular signal transduction [18]. Recently, caveolae have also been reported as important regions that can act as platforms to mediate the pharmacologically reagent-induced signal transduction [19]. In the past decades, Caveolin-1 has been linked to the disease development of PH by numerous studies. It has been reported that mice lacking Caveolin-1 spontaneously developed PH with typical PH features including hyperproliferation and vascular abnormalities, persistent increased PA pressure, and right ventricle hypertrophy [20, 21]. Moreover, diverse functions of Caveolin-1 was further linked to the two major cell types of the pulmonary vasculature, the PASMCs and PA endothelial cells (PAECs) [22]: (a) marked downregulation of Caveolin-1 was reported in PAECs from either experimental PH animal models or PH patients [23]. A short-term administration of a cell-permeable Caveolin-1 peptide [24] or reexpression of Caveolin-1 in endothelium [25] could all prevent or reverse the PH disease pathogenesis; (b) marked upregulation of Caveolin-1 was reported in PASMCs from either experimental PH animal models or PH patients [6, 26]. Moreover, the upregulation of Caveolin-1 in PASMCs contributed to the elevated SOCE and intracellular calcium homeostasis by coupling with the SOCC main component TRPC1 and regulator STIM1 to function as an active complex [6, 8, 26], promoting the proliferation and migration of PASMCs.

Given the fact that BTZ effectively inhibited experimental PH by targeting to the TRPC-SOCE-[Ca^2+^]_i_ signaling axis in PASMCs [13], in this study, we further showed that short-term (3-, 6-, and 12-hour) treatments of BTZ did not alter the Caveolin-1 expression, but a long-term treatment (24- and 60-hour) of BTZ induced obvious decrease in Caveolin-1 expression at both mRNA and protein levels, suggesting that other than TRPC1 and TRPC6, BTZ can also affect the expression of Caveolin-1, another core SOCC mediator in PASMCs. To further determine whether the BTZ-induced downregulation in caveolin-1 protein is completely due to the inhibition on Caveolin-1 transcription, we introduced the protein synthesis inhibitor CHX to rule out the influence of protein production and only focused on the protein degradation process. Our data showed that in the presence of CHX, treatment of BTZ brought further decline in Caveolin-1 protein level and fastened the protein degradation of Caveolin-1. As pointed out in Figure 4, upon blockage of protein synthesis by CHX, BTZ treatment leads to an accelerated time-dependent decrease in both Caveolin-1 and p21 protein, suggesting that (a) without the contribution of transcription and protein synthesis, BTZ may induce the activity of lysosomal degradation pathway, leading to accelerated Caveolin-1 protein degradation; (b) with the presence of both protein synthesis inhibitor CHX and UPS inhibitor BTZ, the decreased p21 protein strongly suggests that either p21 can also degrade through other pathways (e.g., the lysosome pathway) besides the well-known UPS pathway, or blockage of UPS pathway by BTZ somewhat activates lysosomal degradation pathway. This is an important question, which deserves further study.

Caveolin-1 protein is known to be degraded through the lysosomal protein degradation pathway. Studies have shown that when the cells are in a special state (e.g., cholesterol perturbation), the caveolae will be decomposed, where Caveolin-1 is dissociated from the fossa and endocytosis as a cargo protein and then undergo early endosome, late endosome, and multivesicular bodies, and finally, reach lysosome for degradation [16]. Surprisingly, our data showed that long-term (24- and 60-hour) treatment of the specific lysosomal inhibitor CHQ not even resulted in Caveolin-1 accumulation, but surprisingly induced a decline in the Caveolin-1 protein, which is due to an inhibition of CHQ on Caveolin-1 mRNA expression, progressively leading to decrease in Caveolin-1 protein. Considering that both BTZ and CHQ can inhibit the PASMC proliferation and PA remodeling by targeting to the SOCE and calcium regulation, these data partially explain the molecular basis underlying this phenomenon. Moreover, a combined treatment of BTZ and CHQ could not bring further suppression on Caveolin-1 expression, baseline [Ca^2+^]_i_, SOCE, and proliferation, likely because of the effects of BTZ on Caveolin-1 transcription and degradation are already strong enough to cover the effects of CHQ on Caveolin-1 mRNA. These data also suggest that Caveolin-1/SOCE/baseline [Ca^2+^]_i_ signaling axis may act as a common pathway of BTZ and CHQ, two classes of proven anti-PH reagents.

In summary, both the ubiquitin-proteasome inhibitor BTZ and the lysosome inhibitor CHQ can suppress the Caveolin-1/SOCE/baseline [Ca^2+^]_i_ signaling axis by targeting to Caveolin-1 expression, although due to different molecular mechanisms: (a) BTZ inhibits the Caveolin-1 expression by both inhibiting the mRNA transcription and promoting the protein degradation, while (b) CHQ inhibits the Caveolin-1 expression by inhibiting the mRNA transcription. These findings expand our understanding how the inhibitors of the two major protein degradation pathways inhibit PH and uncover a common pathway of their functional consequence by targeting the Caveolin-1/SOCE/baseline [Ca^2+^]_i_ signaling axis. To date, although studies have attempted to dig into the specific mechanisms of both ubiquitin-proteasome inhibitor and lysosomal inhibitor against PH therapy [17, 27–29], unfortunately, we still do not fully understand the detail mechanisms. Our findings provided novel insights into the potential crosslink between these two types of major protein degradation pathways. We believe to further figure out the profile change of both ubiquitin-proteasome inhibitor and lysosomal inhibitor will lead to the full evaluation of the common and distinct pathways underlying the therapeutic consequences and contribute to the future optimization of these two types of anti-PH reagents.

## Data Availability

The data used to support the findings of this study are available from the corresponding author upon request.

## References

[1] Sylvester J. T., Shimoda L. A., Aaronson P. I., Ward J. P. T. (2012). Hypoxic pulmonary vasoconstriction. *Physiological Reviews*.

[2] Pullamsetti S. S., Schermuly R., Ghofrani A., Weissmann N., Grimminger F., Seeger W. (2014). Novel and emerging therapies for pulmonary hypertension. *American Journal of Respiratory and Critical Care Medicine*.

[3] Shimoda L. A., Wang J., Sylvester J. T. (2006). Ca2+ channels and chronic hypoxia. *Microcirculation*.

[4] Wang J., Weigand L., Lu W., Sylvester J. T., Semenza G. L., Shimoda L. A. (2006). Hypoxia inducible factor 1 mediates hypoxia-induced TRPC expression and elevated intracellular Ca2+ in pulmonary arterial smooth muscle cells. *Circulation Research*.

[5] Wang J., Shimoda L. A., Weigand L., Wang W., Sun D., Sylvester J. T. (2005). Acute hypoxia increases intracellular [Ca2+] in pulmonary arterial smooth muscle by enhancing capacitative Ca2+ entry. *American journal of physiology Lung cellular and molecular physiology*.

[6] Yang K., Lu W., Jiang Q. (2015). Peroxisome proliferator-activated receptor *γ*-mediated inhibition on hypoxia-triggered store-operated calcium entry. A caveolin-1-dependent mechanism. *American Journal of Respiratory Cell and Molecular Biology*.

[7] Yang K., Zhao M., Huang J. (2018). Pharmacological activation of PPAR*γ* inhibits hypoxia-induced proliferation through a caveolin-1-targeted and -dependent mechanism in PASMCs. *American journal of physiology Cell physiology*.

[8] Pani B., Liu X., Bollimuntha S. (2013). Impairment of TRPC1-STIM1 channel assembly and AQP5 translocation compromise agonist-stimulated fluid secretion in mice lacking caveolin 1. *Journal of Cell Science*.

[9] Yeh Y. C., Parekh A. B. (2015). Distinct structural domains of caveolin-1 independently regulate Ca2+ release-activated Ca2+ channels and Ca2+ microdomain-dependent gene expression. *Molecular and Cellular Biology*.

[10] Sathish V., Abcejo A. J., Thompson M. A., Sieck G. C., Prakash Y. S., Pabelick C. M. (2012). Caveolin-1 regulation of store-operated Ca (2+) influx in human airway smooth muscle. *The European Respiratory Journal*.

[11] Ong H. L., Ambudkar I. S. (2011). The dynamic complexity of the TRPC1 channelosome. *Channels*.

[12] Ibrahim Y. F., Wong C. M., Pavlickova L. (2014). Mechanism of the susceptibility of remodeled pulmonary vessels to drug-induced cell killing. *Journal of the American Heart Association*.

[13] Zhang J., Lu W., Chen Y. (2016). Bortezomib alleviates experimental pulmonary hypertension by regulating intracellular calcium homeostasis in PASMCs. *American journal of physiology Cell physiology*.

[14] Wang X., Ibrahim Y. F., Das D., Zungu-Edmondson M., Shults N. V., Suzuki Y. J. (2016). Carfilzomib reverses pulmonary arterial hypertension. *Cardiovascular Research*.

[15] Xu L., Chen Y., Yang K. (2014). Chronic hypoxia increases TRPC6 expression and basal intracellular Ca2+ concentration in rat distal pulmonary venous smooth muscle. *PLoS One*.

[16] Parton R. G., Howes M. T. (2010). Revisiting caveolin trafficking: the end of the caveosome. *The Journal of Cell Biology*.

[17] Wu K., Zhang Q., Wu X. (2017). Chloroquine is a potent pulmonary vasodilator that attenuates hypoxia-induced pulmonary hypertension. *British Journal of Pharmacology*.

[18] Parton R. G., del Pozo M. A. (2013). Caveolae as plasma membrane sensors, protectors and organizers. *Nature Reviews Molecular Cell Biology*.

[19] Patel H. H., Murray F., Insel P. A. (2008). Caveolae as organizers of pharmacologically relevant signal transduction molecules. *Annual review of pharmacology and toxicology*.

[20] Zhao Y. Y., Liu Y., Stan R. V. (2002). Defects in caveolin-1 cause dilated cardiomyopathy and pulmonary hypertension in knockout mice. *Proceedings of the National Academy of Sciences of the United States of America*.

[21] Razani B., Engelman J. A., Wang X. B. (2001). Caveolin-1 null mice are viable but show evidence of hyperproliferative and vascular abnormalities∗. *The Journal of Biological Chemistry*.

[22] Mathew R. (2014). Pathogenesis of pulmonary hypertension: a case for caveolin-1 and cell membrane integrity. *American Journal of Physiology Heart and Circulatory Physiology*.

[23] Mathew R., Huang J., Shah M., Patel K., Gewitz M., Sehgal P. B. (2004). Disruption of endothelial-cell caveolin-1alpha/raft scaffolding during development of monocrotaline-induced pulmonary hypertension. *Circulation*.

[24] Jasmin J. F., Mercier I., Dupuis J., Tanowitz H. B., Lisanti M. P. (2006). Short-term administration of a cell-permeable caveolin-1 peptide prevents the development of monocrotaline-induced pulmonary hypertension and right ventricular hypertrophy. *Circulation*.

[25] Murata T., Lin M. I., Huang Y. (2007). Reexpression of caveolin-1 in endothelium rescues the vascular, cardiac, and pulmonary defects in global caveolin-1 knockout mice. *The Journal of Experimental Medicine*.

[26] Patel H. H., Zhang S., Murray F. (2007). Increased smooth muscle cell expression of caveolin-1 and caveolae contribute to the pathophysiology of idiopathic pulmonary arterial hypertension. *FASEB Journal*.

[27] Dunmore B. J., Drake K. M., Upton P. D., Toshner M. R., Aldred M. A., Morrell N. W. (2013). The lysosomal inhibitor, chloroquine, increases cell surface BMPR-II levels and restores BMP9 signalling in endothelial cells harbouring BMPR-II mutations. *Human Molecular Genetics*.

[28] Long L., Yang X., Southwood M. (2013). Chloroquine prevents progression of experimental pulmonary hypertension via inhibition of autophagy and lysosomal bone morphogenetic protein type II receptor degradation. *Circulation Research*.

[29] Wade B. E., Zhao J., Ma J., Hart C. M., Sutliff R. L. (2018). Hypoxia-induced alterations in the lung ubiquitin proteasome system during pulmonary hypertension pathogenesis. *Pulmonary circulation*.

